# Supplementary material for: Neuronal activation sequences in lateral prefrontal cortex encode visuospatial working memory during virtual navigation
Source: Nat Commun. 2024 May 25;15:4471. doi: 10.1038/s41467-024-48664-9 (PMC11127969; doi:10.1038/s41467-024-48664-9)
Supplement: Supplementary file 1 — Supplementary Information [file 41467_2024_48664_MOESM1_ESM.pdf]

1 **Supplementary Figures**

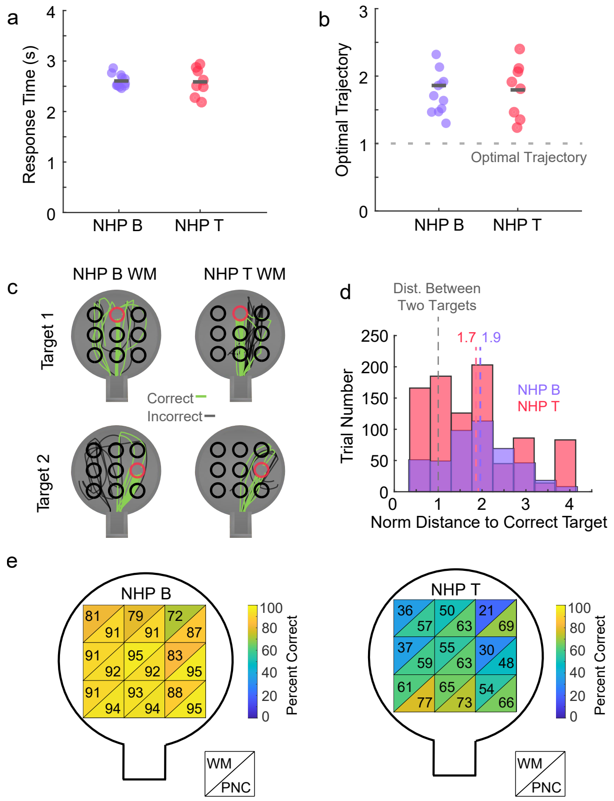

Fig. S1. Behaviour — Task Performance (**A**) Response time for correct trials. Black lines indicate the median values for each animal. Dots represent data for each session. (**B**) Optimal trajectory for correct trials. Calculated by real trajectory length/ Euclidean distance between the start location and target location. The dashed gray line indicates the optimal value of 1. Black lines represent median values and dots represent data for each session. (**C**) Example animal trajectories for two target locations. Red circles indicate the target, green lines represent correct trials and gray lines represent incorrect trials. (**D**) Distance from target for incorrect trials. Calculated as the Euclidean distance from the animal's end location to the correct target location. Since 'Unreal' units are arbitrary, distance values are normalized by the distance (in 'Unreal' units) between two targets. A normalized distance of 1 indicated by the dashed gray line is the distance between two target centers. Dashed purple and red lines represent median values. (**E**) Percent of correct trials to each target location, split by subject and task (WM compared to perception-navigation control (PNC)). Targets are depicted as arranged in the virtual circular arena. Panels 'a' and 'b' have been adapted from Roussy et al., 2022. For panels a,b,d N=20 WM sessions. For panel e, N=17 WM sessions.

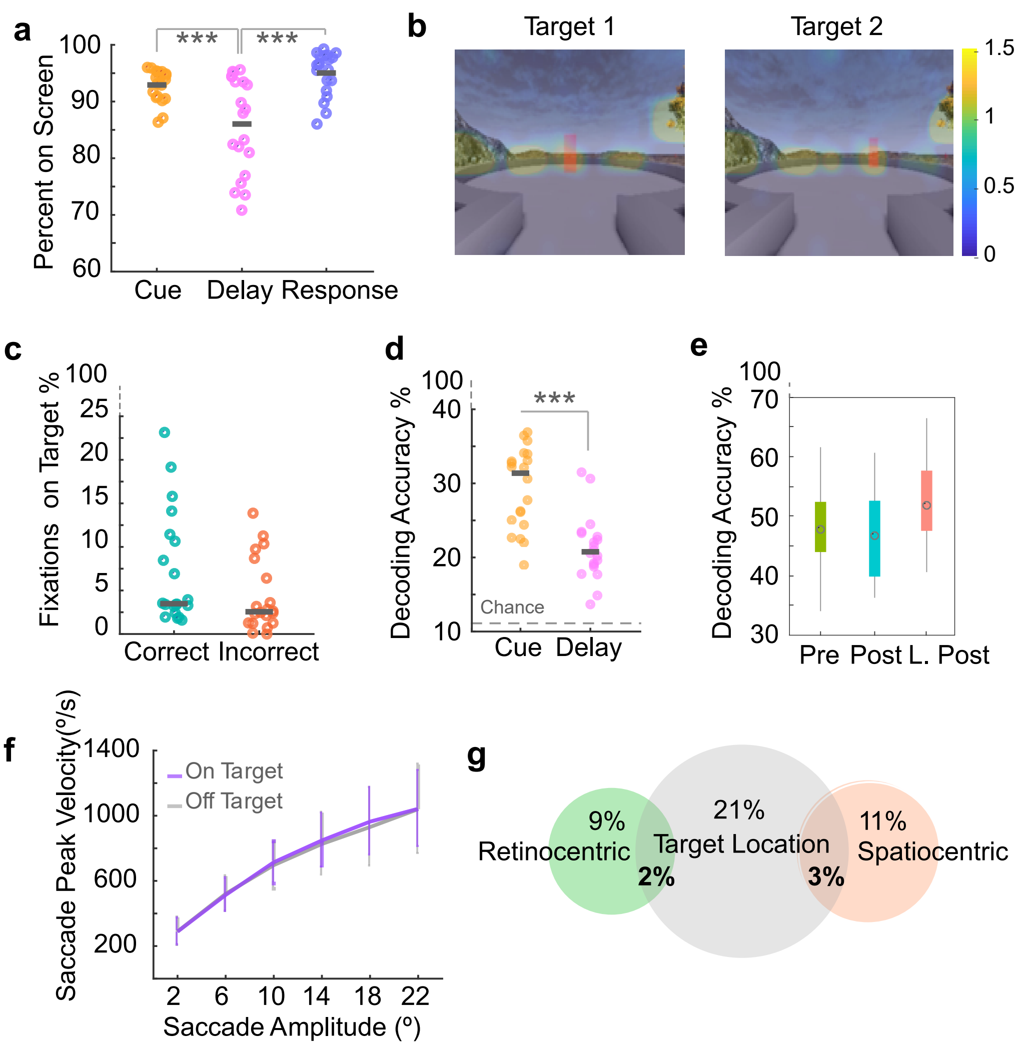

Fig. S2. Behaviour — Eve Movement (**A**) Percent of eye data points falling on screen for different task epochs. Black lines represent mean values for each group and dots represent data from each session. (**B**) Heat maps of eye fixation position on screen during delay for two target examples. Eye fixation is concentrated in task relevant areas on screen but is not primarily concentrated on the target location.(**C**) Percentage of total fixations during the delay epoch that land within the target location for correct and incorrect trials. The black lines represent median values and the dots represent data for each session. (**D**) Decoding accuracy for predicting target location from eye fixation location during the cue and delay epochs. The black lines represent median values and the dots represent data for each session. The gray line indicates chance performance for 9 classes. (p=4.2e-5, Wilcoxon rank sum)(**E**) Decoding accuracy for decoding target location from eye fixation position during the delay period for different ketamine injection periods. Decoding is shown for 3 classes (33.33% chance). The gray circles indicate median values. (**F**) Main sequence values during the delay period for saccades that fall on and off target location.(**G**) Proportion of neurons tuned for saccade landing position in retinocentric and spatiocentric reference frames as well as the proportion of neurons tuned for target location during the delay period. Overlapping sections indicate neurons that are selective for both remembered target location and saccade position. Panels 'f' and 'g' have been adapted from Roussy et al, 2021. Panels 'b', 'c', 'd', and 'e' have been adapted from Roussy et al., 2022. N=20 WM sessions for all panels. *p < 0.05, **p < 0.01, ***p < 0.001. See statistics table for additional details.

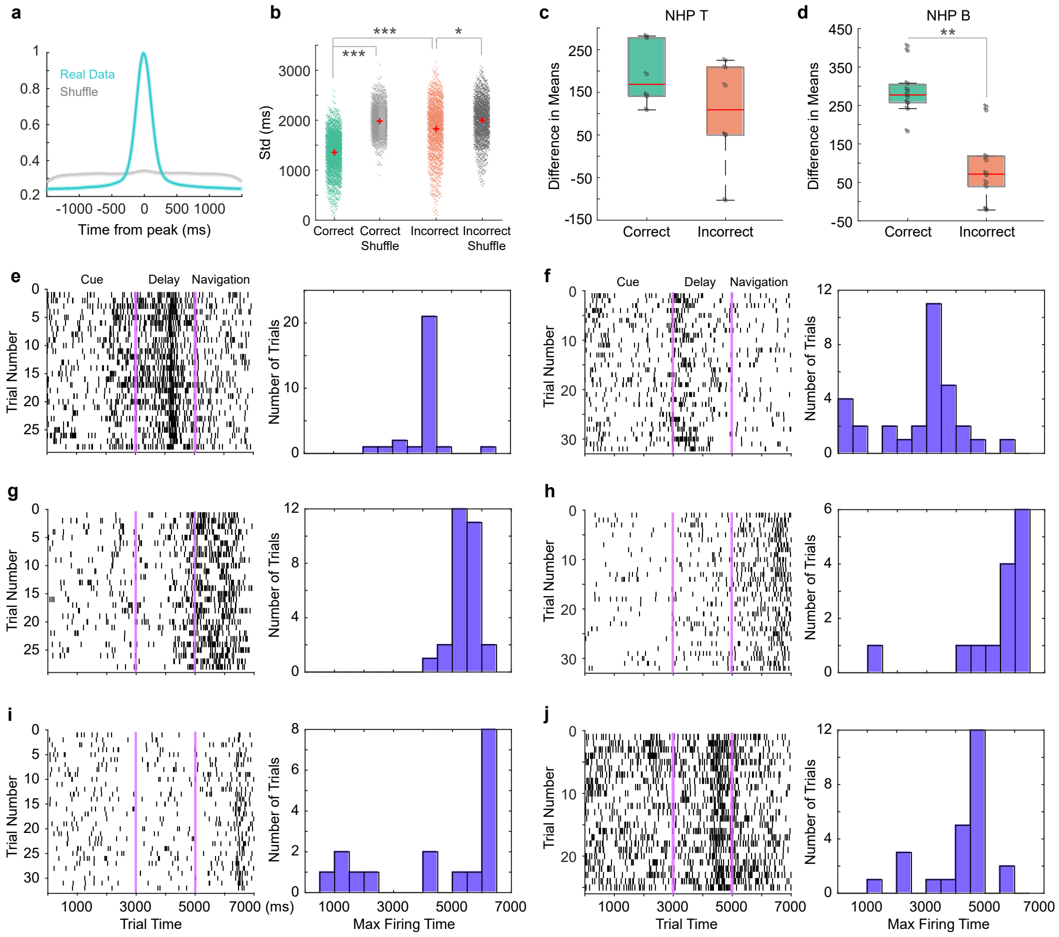

Fig. S3. Time-Consistent Neurons (**A**) Alignment of peak firing times for all neurons (blue) compared to shuffled peak firing times (grey). Here, we circularly shifted the normalized SDF of each neuron in each trial so that their maximum firing times were aligned (x axis = 0). We then averaged the aligned SDFs to produce the blue distribution, which demonstrates the timescale of these elevations in firing. The grey distribution results from first shuffling the spikes of the neurons before repeating this procedure. (**B**) Trial-trial standard deviation in max firing time for each neuron across sessions during correct and incorrect trials and for shuffled correct and incorrect data. The red crosses indicate group means. (**C**) Difference in means between real and shuffled distributions of standard deviations of max firing times across trials. Presented for correct and incorrect trials for NHP T. The red lines represent median values. Dots represent data for N=6 individual sessions. (**D**) Difference in means between real and shuffled distributions of standard deviations of max firing times across trials. Presented for correct and incorrect trials for NHP B. The red lines represent median values. Dots represent data for N=11 individual sessions. ($p=0.002$, Wilcoxon signed rank test) (**E-J**) Example single neurons. Left column represents the activity of one example neuron over trial time over all trials of a certain condition. Pink lines separate task epochs. Right column shows a histogram of max firing times per trial. *$p < 0.05$, **$p < 0.01$, ***$p < 0.001$. See statistics table for additional details.

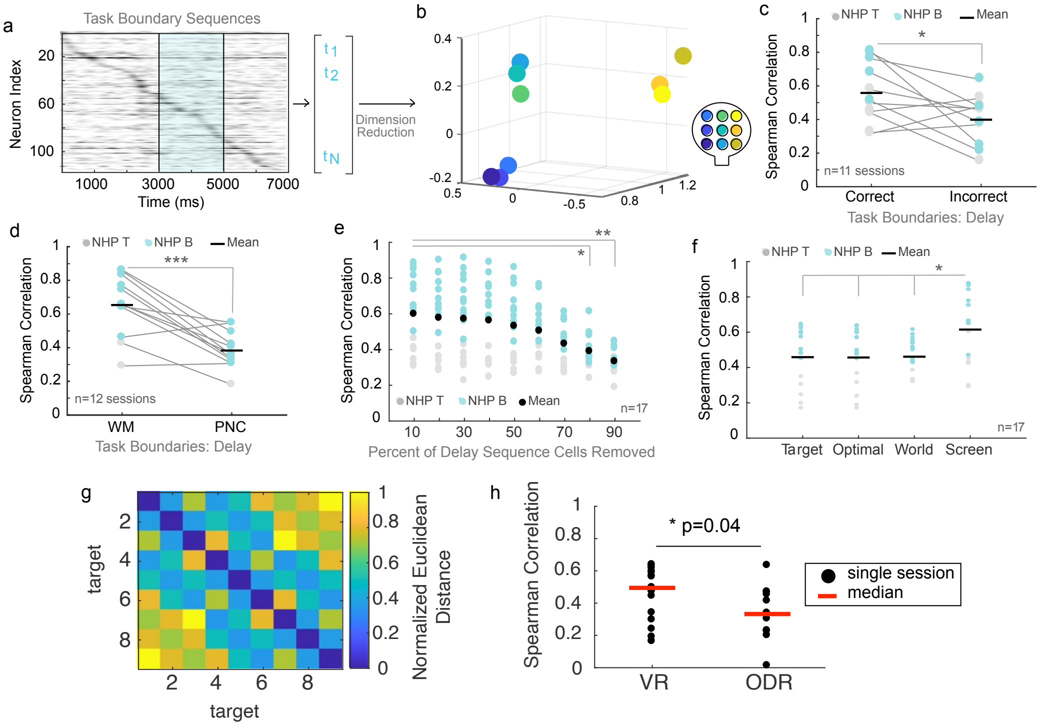

Fig. S4. Correlation Controls (**A**) Depiction of single-trial sequence representation using the delay epoch boundaries defined by the task structure. (**B**) The resulting sequence centroids for one recording session, depicted alongside the target locations in the VR arena. (**C**) Correlations between delay sequence centroids and visuospatial trajectories for sessions with both correct and incorrect trials for all 9 target locations. (Note that panels a-c correspond to Fig. 3a-c, using task epoch boundaries as opposed to neural epoch boundaries.) (p=0.04, Paired T-Test, N=17 WM sessions) (**D**) Correlations between delay sequence centroids and visuospatial trajectories for correct trials during the WM task compared to the perception-navigation control task (PNC). (p=3.4e-4, Paired T-Test, 12 matched WM and PNC sessions) (**E**) Ablation test, reproducing the correlation analysis for the WM memory period sequences after randomly removing a percentage of cells. Here, each dot represents the mean of 10 iterations for a single recording session. Black dots represent mean values across sessions. (p=4.9e-6, 1-way ANOVA with multiple comparisons corrections - see statics table for details) (**F**) Comparison of correlations between neural sequence centroids and different task variables: “Target” denotes the locations of the 9 targets; “Optimal” denotes the set of shortest paths from the start location to each target location; “World” denotes the set of trajectories in 3D virtual space the subjects took while navigating to the targets; “Screen” denotes the world trajectories as they appear through a perspective projection onto the computer screen. (p=0.03, 1-way ANOVA with multiple comparisons correction, N=17 WM sessions, see statistics table for details.) (**G**) To be sure the increased correlation between sequences and behavior in our VR task compared to the ODR task (Fig. 4f) was not a result of the clustered behavioral matrix in VR (see for example Fig. 3g), we repeated the analysis using the matrix of distances between target locations, plotted here. (**H**) The resulting correlations remain significantly higher in VR compared to ODR. *p < 0.05, **p < 0.01, ***p < 0.001. See statistics table for additional details.

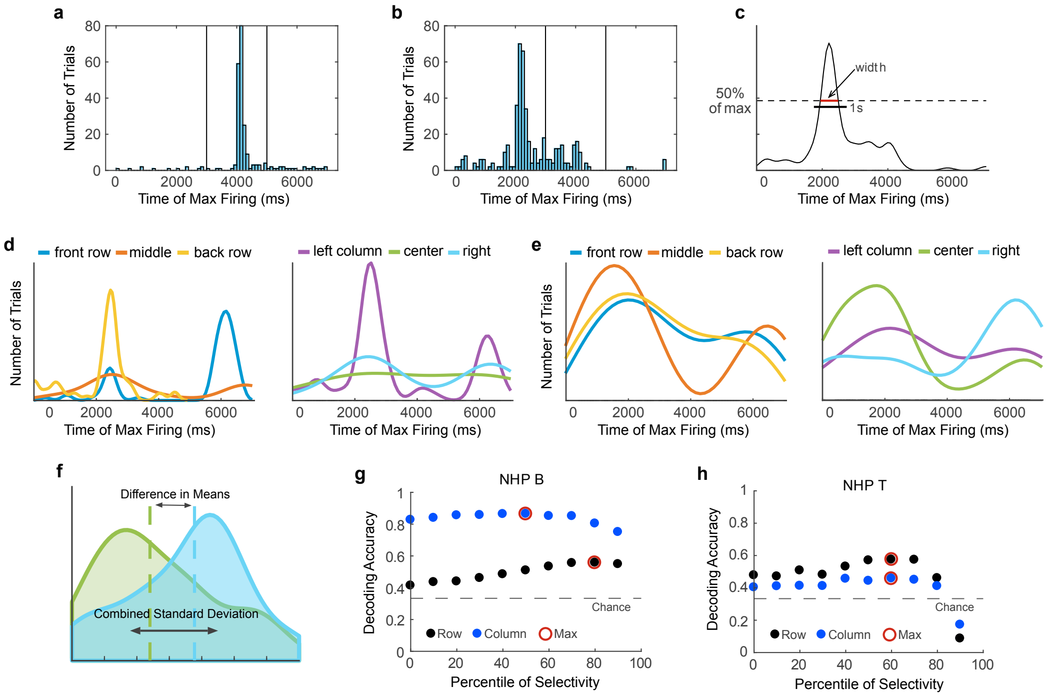

Fig. S5. Cell Selectivity (**A-B**) Two example time-selective cells. The histograms plot the times at which these cells contribute to the single-trial sequence across trials of all 9 target conditions. Vertical black lines indicate the start and end of the memory period. (**C**) Determining time selectivity. The distribution of firing times is plotted for the example cell in 'b'. A cell is considered time selective if the width (red line) of the distribution at 50% of its maximum is less than a threshold (black line). Threshold values were 1s for NHP B (as depicted), and 2s for NHP T. (**D**) Example row-selective cell. Left: distributions of max firing times for targets in each row. We see distinct peaks in the yellow and blue distributions, i.e., this neuron contributes to the sequence around 3000ms for targets in the front row, and around 6000ms for targets in the back row. Right: distributions of max firing times for targets in each column. This cell shows no clear separation in firing time by column. (**E**) Same as 'd', for a column-selective cell. Here, we see separation between the firing times for the center and right columns (green and blue distributions). (**F**) Schematic: the effect size between each pair of rows (or each pair of columns) is computed, as the difference in means of the two distributions divided by the combined standard deviation. (**G,H**) Thresholds for selectivity for each subject, determined by best decoding accuracy. For each percentile of selectivity (maximum effect size), sub-sequences of cells exceeding this percentile for row and column selectivity were used to decode row and column respectively. Decoding accuracy is plotted as a function of selectivity. Maximum decoding accuracy is used to define the thresholds for row and column selectivity used for each subject. These thresholds determine the cells which contribute to the row and column decoding in Fig. 4d.

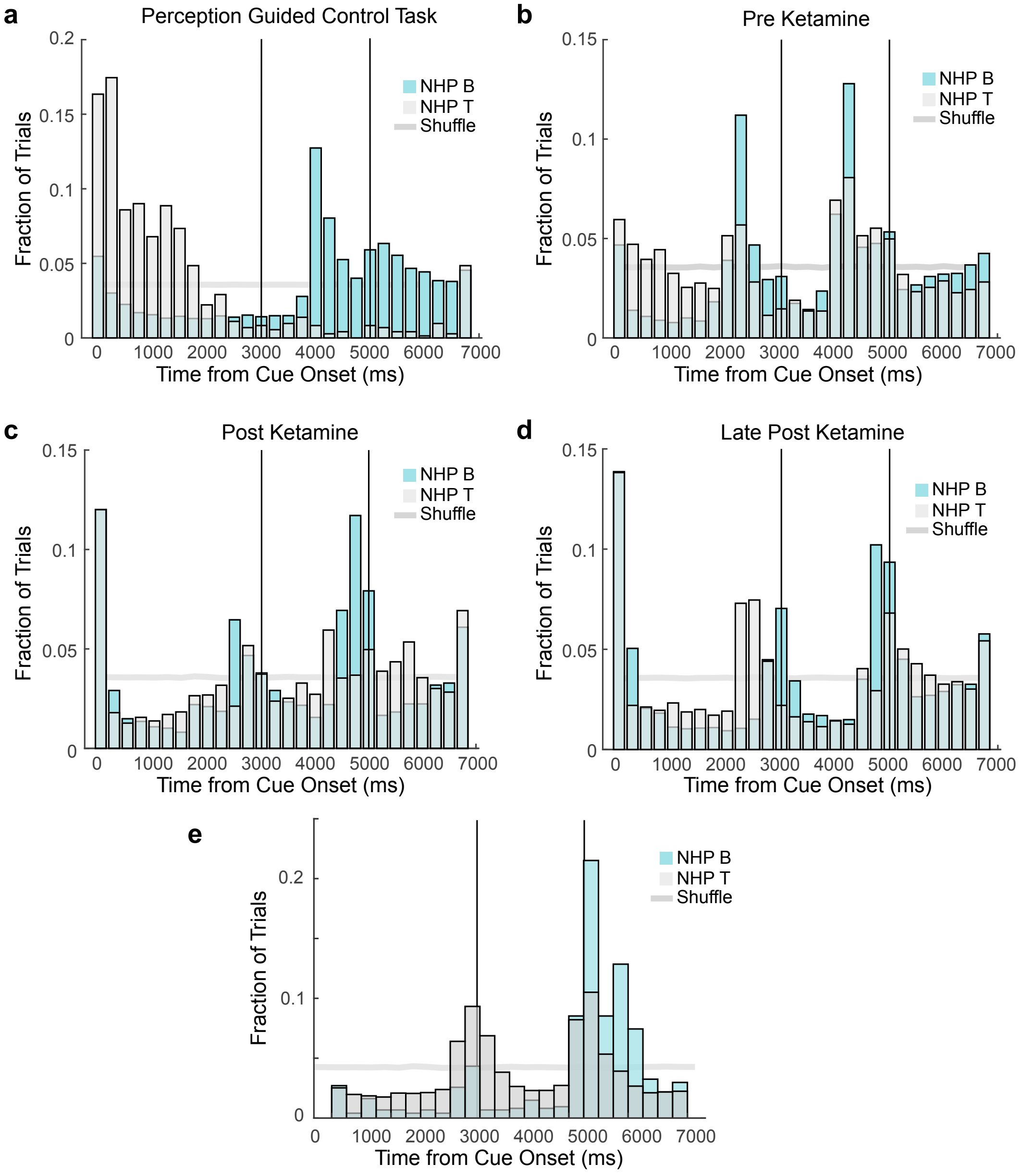

Fig. S6. Neural Boundaries (**A-D**) Times at which time-selective cells contribute to single-trial sequences across trials of all 9 target conditions. The shuffle control was created by randomly shuffling spike times of time-selective cells within trials. (**A**) Neural boundaries are not present during the perception control task, in which WM is not required. (**B**) Neural boundaries prior to ketamine administration.(**C**) Neural boundaries post ketamine administration. (**D**) Neural boundaries 1 hour after ketamine administration. (**E**) Neural boundaries during WM task - same as Fig. 3e, but using the same threshold of 1500ms for time-selective cells for each subject.

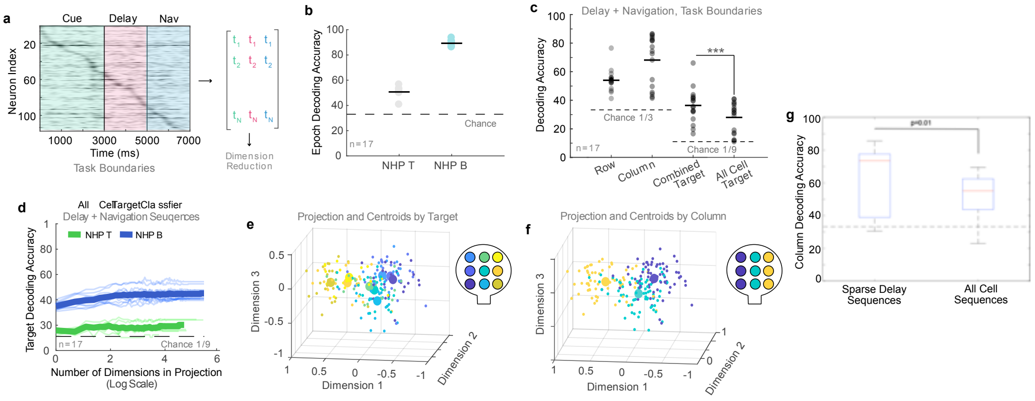

Fig. S7. Decoding Analyses (**A**) Depiction of single-trial sequence representations for cue, delay, and navigation epochs, using the task epoch boundaries, as marked by vertical black lines. (**B**) Epoch decoding accuracy using task boundaries. (See Fig 4c for accuracy using neural epoch boundaries.) (**C**) Row, column and target decoding accuracy using the task boundary delay and navigation sequences. (See Fig 4d for accuracy using neural boundaries.)(**D**) Target decoding accuracy as a function of the number of dimensions included in the projection, depicted alongside corresponding targets in the virtual arena. (**E**) Example projection coloured by target location. (**F**) Example projection coloured by target column, depicted alongside corresponding targets in the virtual arena. (**G**) Decoding performance of delay sequences as defined throughout the text, where each cell participates in the sequence only once per trial (and only a subset of cells participate during the delay), compared to sequences where each cell contributes during the delay period at the time of max firing during the delay. *p < 0.05, **p < 0.01, ***p < 0.001. See statistics table for additional details.

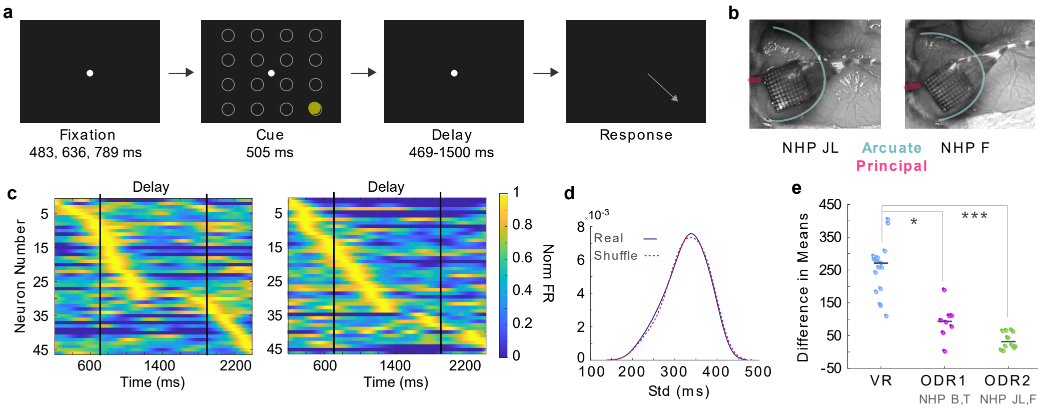

Fig. S8. Temporal organization of neural activity during ODR task (**A**) Depiction of ODR task with 16 targets. (**B**) Surgical images showing location of Utah arrays implanted in left LPFC of NHP JL and NHP F. (**C**)Two trial examples of simultaneously recorded population activity. Normalized firing rate for each neuron is arranged by max firing time. Task timing for delay epoch is marked by black lines. (**D**) Real and shuffled distributions of max firing time trial-to-trial standard deviations for an example session. (**E**) Difference in the means between real and shuffled distributions for the virtual reality task and the ODR task. The VR task and ODR1 task are from NHP B and NHP T, while ODR2 is from NHP JL and NHP F. Gray lines indicate median values and dots represent data per session. *$p < 0.05$, **$p < 0.01$, ***$p < 0.001$. See statistics table for additional details.

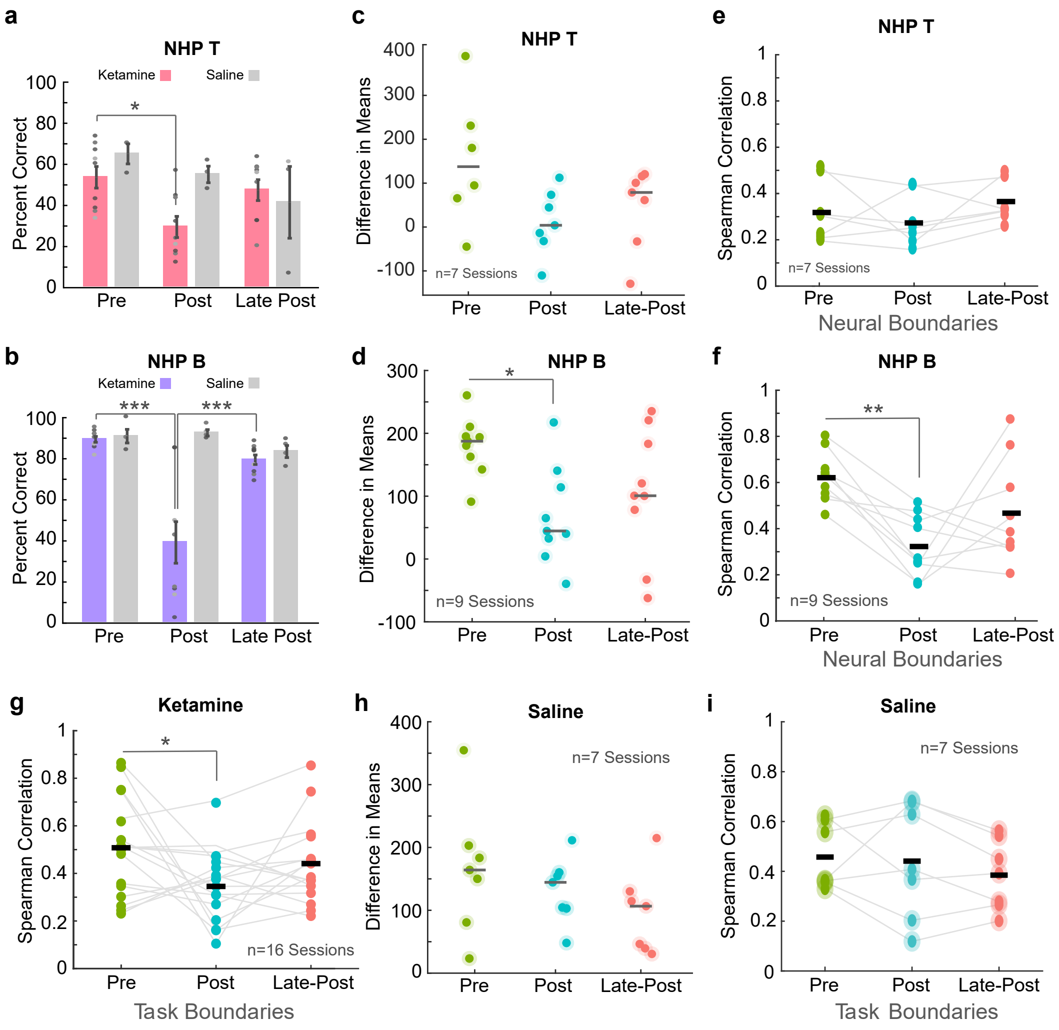

Fig. S9. Ketamine and Saline Control Analyses (**A**) Percent of correct trials for ketamine and saline sessions over injection periods for NHP T. Dots represent data for individual sessions and error bars are SEM. (**B**) Percent of correct trials for ketamine and saline sessions over injection periods for NHP B. Dots represent data for individual sessions and error bars are SEM. (**C**)Difference in the means between real and shuffled distributions of standard deviation values for neuron max firing time between trials. Presented for each ketamine injection period for NHP T. Dots represent data for each session. Black lines indicate the mean across sessions. (**D**) Difference in means between real and shuffled distributions for each ketamine injection period for NHP B. Dots represent data for each session. Black lines indicate the mean across sessions. (**E**) Correlation between the distances between condition cluster centroids and the distances between visuospatial trajectories, using neural boundary delay sequences. Correlation values are presented for ketamine injection periods for NHP T. Dots represent data per session, and lines indicate injection periods from the same session. Black lines indicate the mean across sessions. (**F**) Correlation between the distances between condition cluster centroids and the distances between visuospatial trajectories, using neural boundary delay sequences. Correlation values are presented for ketamine injection periods for NHP B. Dots represent data per session, and lines indicate injection periods from the same session. Black lines indicate the mean across sessions. (**G**) Correlation values for ketamine injection periods for both subjects using delay sequences with task epoch boundaries. Black lines indicate the mean across sessions. (See Fig. 5e for correlations using neural boundaries.)(**H**) Difference in mean values between real and shuffled distributions of max firing time standard deviations for saline injection periods. Dots represent data for each session and black lines indicate the mean across sessions (NHP T and NHP B combined). (**I**) Correlation between the distance between condition cluster centroids and distance between target trajectories. Correlation values are presented for saline injection periods for both animals combined, with balck lines indicating the mean across sessions. *p < 0.05, **p < 0.01, ***p < 0.001. See statistics table for additional details.

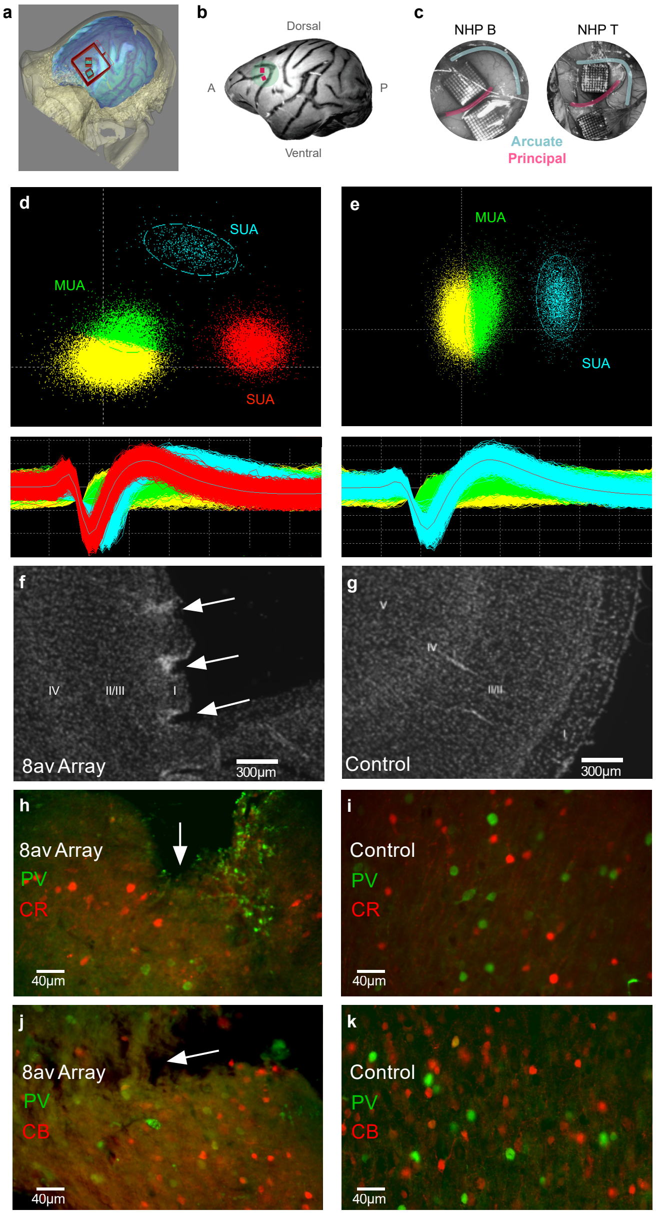

Fig. S10. Neural Recording Setup (**A**) Graphic of presurgical planning procedure showing 3D reconstructed skull and brain based on CT and MRI scans. Electrode array positioning is illustrated in blue with a red outline. The craniotomy is outlined by the larger red box. (**B**) 3D modeled brain with electrode array placement in pink. (**C**)Surgical images of array implantation in NHP B and NHP T. (**D**) Example of spike sorting for one electrode channel. Upper panel represents PCA space and the lower panel represents individual threshold crossing event waveforms. The blue and red clusters represent what we would classify as a single unit. The green cluster would be classified as a multiunit. (**E**) Example of spike sorting for one electrode channel. The blue cluster would represent a single unit. The green cluster would represent multiunit activity. This figure is modified from Roussy et al., 2021). (**F,G**) f,g, Histology showing cortical layers relative to array location (f), compared to healthy adjacent cortex as a control (g). Arrows indicate array placement. (**H,I**) Histology staining for Parvalbumin (green) and Calretenin (red) in the implanted subject (h) compared to healthy adjacent cortex as a control (i). Arrows indicate array placement. (**J,K**) Histology staining for Parvalbumin (green) and Calbindin (red) in the implanted subject (j) compared to healthy adjacent cortex as a control (k). Arrows indicate array placement.

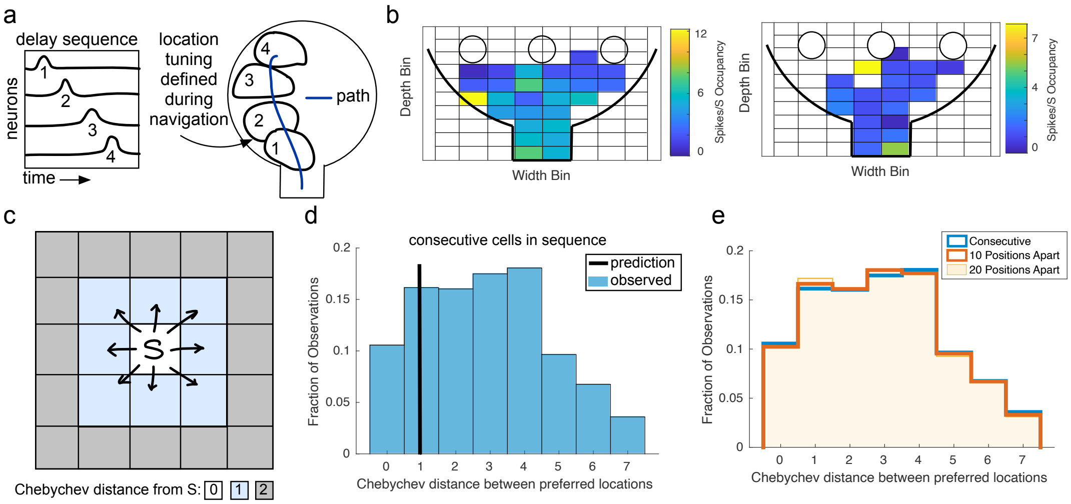

Fig. S11. Sequences are not direct representations of paths. (**A**) Schematic of the possibility that delay NASs represent the sequential activation (in time) of neurons tuned for sequential locations (in space) during navigation.(**B**) To test the possibility that sequences represent a bump moving through mnemonic space, we first defined preferred locations for each cell based on maximum spiking activity during navigation. To do so, we binned the VR arena into a "checkerboard" (grid lines) and determined whether cells fired preferentially when the animal was navigating through specific bins. We note that for this analysis we considered only the targets in the first row (white circles). Here, firing fields of two example cells during navigation with bins colored by the number of spikes per second of occupancy time. (**C**) Schematic of the Chebychev or "king's chessboard" distance metric used in the following panels. If sequence activations trace out a trajectory through mnemonic space, the Chebychev distance between consecutive cells should be 1 (prediction). (**D**) The observed distances between preferred locations for consecutive cells in the delay sequence, pooled across all sessions (observed). (**E**) The same measure repeated for cells that participate in the sequence 10 positions apart (red line) and 20 positions apart (yellow shaded area). The overlap suggests there is no relationship between the position of cells in a sequence and the distance between their preferred locations.

MAIN TEXT FIGURE STATISTICS						
**Fig.**	**Subj.**	**Data**	**Stat Test**	**Comparison**	**Stat-Value**	**P-Value**
**1e**<br>**Percent of Correct Trials**	NHP B, NHP T	20 WM Sessions			NHP B<br>mean = 87%<br>median = 85%<br>NHP T<br>mean = 57%<br>median = 56%	
**2g**<br>**Difference in Means**	NHP B, NHP T	17 WM Sessions	Wilcoxon Signed Rank Test	Correct and incorrect trials	*Rank* = 161<br>Correct<br>mean = 253.98<br>median = 270.93<br>Incorrect<br>mean = 93.69<br>median = 71.43	0.001
**3h**<br>**Correlation Analysis**	NHP B, NHP T	11 WM Sessions (correct and incorrect trials for all 9 targets)	Paired T-Test	Correlation between average trajectories and centroids defined by correct vs incorrect trial sequences	t = 3.65<br>Correct<br>**mean** = 0.52<br>median = 0.50<br>Incorrect<br>**mean** = 0.27<br>median = 0.30	0.0040
**4c**<br>**Epoch Decoding**	NHP B, NHP T	17 WM sessions	T-Test	Decoder vs chance (33%)	t = 9.53<br>Decoding accuracy:<br>**mean** = 76%<br>median = 87%	5.3779e-08

MAIN TEXT FIGURE STATISTICS						
**Fig.**	**Subj.**	**Data**	**Stat Test**	**Comparison**	**Stat-Value**	**P-Value**
**4d**<br><br>**Row Decoding**	NHP B, NHP T	17 WM sessions	T-Test	Decoder vs chance (33%)	t = 13.74<br><br>Decoding accuracy:<br>**mean** = 56%<br>median = 55%	2.8203e-10
**4d**<br><br>**Column Decoding**	NHP B, NHP T	17 WM sessions	T-Test	Decoder vs chance (33%)	t = 7.65<br><br>Decoding accuracy:<br>**mean** = 71%<br>median = 83%	9.9221e-07
**4d**<br><br>**Target decoding**	NHP B, NHP T	17 WM sessions	Paired T-Test	Combined row + column decoder vs "all cell" sequence decoder	t = 6.46<br><br>Combined:<br>**mean** = 40%<br>median = 43%<br><br>All Cell:<br>**mean** = 31%<br>median = 34%	7.79e-06
**4f**<br><br>**Correlation Analysis**	NHP B, NHP T	17 WM sessions<br><br>11 ODR Sessions	Rank Sum	VR compared to ODR	z=3.0775<br><br>VR:<br>mean =0.61<br>**median** =0.64<br><br>ODR:<br>mean =0.34<br>**median** = 0.333	0.0021

4

MAIN TEXT FIGURE STATISTICS						
**Fig.**	**Subj.**	**Data**	**Stat Test**	**Comparison**	**Stat-Value**	**P-Value**
**5b**	NHP B,<br>NHP T	16 WM sessions	1-way ANOVA<br><br>Tukey-Kramer Post Hoc	Sequences compared to persistent (with different criteria for persistence)	F=28.57<br><br>Sequences:<br>**mean** =0.31<br>median =0.34<br><br>p=0.05<br>**mean** =0.17<br>median =0.16<br><br>p=0.1<br>**mean** =0.19<br>median =0.18<br><br>p=0.2<br>**mean** =0.15<br>median =0.15<br><br>p=0.3<br>**mean** =0.15<br>median =0.14	3.79E-14
				Seq - P=0.05		0.00
				Seq - P=0.1		0.00
				Seq - P = 0.2		0.00
				Seq - P = 0.3		0.00
				P=0.05-0.1		0.78
				P=0.05-0.2		0.88
				P=0.05-0.3		0.62
				P=0.1-0.2		0.18
				P=0.1-0.3		0.06
				P=0.2-0.3		0.98

MAIN TEXT FIGURE STATISTICS						
**Fig.**	**Subj.**	**Data**	**Stat Test**	**Comparison**	**Stat-Value**	**P-Value**
**5d**	NHP B,<br>NHP T	5 ODR sessions	1-way ANOVA<br><br>Tukey-Kramer Post Hoc	Sequences compared to persistent (with different criteria for persistence)<br><br>Seq - P=0.05<br>Seq - P=0.1<br>Seq - P = 0.2<br>Seq - P = 0.3<br>P=0.05-0.1<br>P=0.05-0.2<br>P=0.05-0.3<br>P=0.1-0.2<br>P=0.1-0.3<br>P=0.2-0.3	F=6.67<br><br><u>Sequences:</u><br>**mean** =0.23<br>median =0.18<br><br><u>p=0.05</u><br>**mean** =0.45<br>median =0.46<br><br><u>p=0.1</u><br>**mean** =0.45<br>median =0.44<br><br><u>p=0.2</u><br>**mean** =0.46<br>median =0.46<br><br><u>p=0.3</u><br>**mean** =0.15<br>median =0.45	0.001<br><br>0.00<br>0.00<br>0.00<br>0.00<br>1.00<br>0.99<br>1.00<br>0.99<br>0.99<br>0.99

MAIN TEXT FIGURE STATISTICS						
**Fig.**	**Subj.**	**Data**	**Stat Test**	**Comparison**	**Stat-Value**	**P-Value**
**6b**<br>**Percent correct**	NHP B, NHP T	18 Ketamine WM sessions<br><br>4 Ketamine perception sessions	Kruskal Wallis<br><br>Tukey-Kramer Post Hoc	WM injection period<br><br>Perception Injection Period	*H*(2,51) = 18.75<br><br>Pre, early post<br><br>Pre, late-post<br><br>Early-post, late-post<br><br>*H*(2,9) = 0.13<br><br><u>Pre-injection WM</u> mean = 72% **median** = 77%<br><br><u>Early Post-Injection WM</u> mean = 34% **median** = 28%<br><br><u>Late Post-Injection WM</u> mean = 63% **median** = 66%<br><br><u>Pre-Injection Perception</u> mean = 79% **median** = 84%	8.49E-05<br><br>7.7E-05<br><br>0.54<br><br>0.008<br><br>0.94

MAIN TEXT FIGURE STATISTICS						
**Fig.**	**Subj.**	**Data**	**Stat Test**	**Comparison**	**Stat-Value**	**P-Value**
					Early Post-Injection Perception<br>mean = 84%<br>**median** = 90%<br><br>Late Post-Injection Perception<br>mean = 85%<br>**median** = 93%	

MAIN TEXT FIGURE STATISTICS						
**Fig.**	**Subj.**	**Data**	**Stat Test**	**Comparison**	**Stat-Value**	**P-Value**
**6c**<br>**Difference in Means**	NHP B, NHP T	16 Ketamine WM sessions	Kruskal Wallis	Injection period	*F(2,44)* = 10.59	0.005
			Tukey Kramer Post Hoc	Pre, Early post-injection		0.004
				Pre, Late post-injection		0.07
				Early post-injection, late post-injection		0.59
					Pre-injection mean = 161.9 **median** = 171.6<br>Early Post-Injection mean = 40.9 **median** = 40.2<br>Late Post-Injection mean = 71.99 **median** = 100.4	

MAIN TEXT FIGURE STATISTICS						
**Fig.**	**Subj.**	**Data**	**Stat Test**	**Comparison**	**Stat-Value**	**P-Value**
**6d**<br><br>**Correlation**	NHP B, NHP T	17 Ketamine WM sessions	1-way ANOVA<br><br>Tukey Kramer Post Hoc	Injection period<br><br>Pre, Early post-injection<br><br>Pre, Late post-injection<br><br>Early post-injection, late post-injection	*F(2,45) = 4.93*<br><br><u>Pre-injection</u> **mean** = 0.48 median = 0.52<br><br><u>Early Post-Injection</u> **mean** =0.26 median = 0.22<br><br><u>Late Post-Injection</u> **mean** = 0.39 median = 0.34	0.0041<br><br>0.0029<br><br>0.3410<br><br>0.1030

SUPPLEMENTARY FIGURE STATISTICS						
**Fig.**	**Subj.**	**Data**	**Stat Test**	**Comparison**	**Stat-Value**	**P-Value**
**S1d**<br><br>**Distance to correct target**	NHP B, NHP T	20 WM sessions			<u>NHP B</u><br>mean=1.912<br>**median**=1.87<br><br><u>NHP T</u><br>mean=1.81<br>**median**=1.68	
**S2a**<br><br>**Percent Eyes on Screen**	NHP B, NHP T	20 WM sessions	1-way ANOVA<br><br>Tukey Kramer Post Hoc	Epoch<br><br>Cue-Delay<br>Cue-Navigation<br>Delay-Navigation	*F*(2,57) = 13.96<br><br><u>Cue</u><br>**mean**= 93%<br>median = 94%<br><br><u>Delay</u><br>**mean** = 86%<br>median = 88%<br><br><u>Navigation</u><br>**mean** = 95%<br>median = 97%	1.16E-05<br><br>0.0008<br>0.47<br>1.4E-05
**S2c**<br><br>**Percent Fixations on Target**	NHP B, NHP T	20 WM sessions	Wilcoxon Rank Sum<br><br>Paired T-Test	Correct-incorrect	*Rank* = 482<br><br>t(38) = 1.66<br><br><u>Correct</u><br>mean = 7.2%<br>**median** = 3.5%<br><br><u>Incorrect</u><br>mean = 4%<br>**median** = 2.6%	0.05<br><br>0.1

SUPPLEMENTARY FIGURE STATISTICS						
**Fig.**	**Subj.**	**Data**	**Stat Test**	**Comparison**	**Stat-Value**	**P-Value**
**S2d**<br>**Decoding Accuracy**	NHP B, NHP T	20 WM sessions	Wilcoxon Rank Sum	Cue and delay epochs	*Rank = 562*<br>Cue<br>mean = 29.3<br>**median** = 31.4<br>Incorrect<br>mean = 21.2<br>**median** = 20.8	4.2E-05
**S2e**<br>**Main Sequence**	NHP B, NHP T	20 WM sessions	T-Test	Saccade on and off-target		0 time bins, $p < 0.05$
					Cohen's d	0 time bins > 0.2
**S3b**<br>**Trial-Trial Std**	NHP B, NHP T	11 WM sessions	1-way ANOVA	Correct, incorrect, correct shuffle, and incorrect shuffle	$f(3,40) = 79.1$	7.3E-17
			Tukey Kramer Post-Hoc	Correct-correct shuffle		3.77E-09
				Correct-incorrect		3.78E-09
				Correct-incorrect shuffle		3.77E-09
				Correct shuffle-incorrect		0.02
				Correct shuffle-incorrect shuffle		0.99
				Incorrect-incorrect shuffle		0.01

SUPPLEMENTARY FIGURE STATISTICS						
**Fig.**	**Subj.**	**Data**	**Stat Test**	**Comparison**	**Stat-Value**	**P-Value**
**S3c** **Difference in Means**	NHP T	6 WM sessions	Wilcoxon Signed Rank Test	Correct and incorrect	*Rank = 14* Correct mean = 191.12 **median** =168.74 Incorrect mean = 99.71 **median** = 108.98	0.56
**S3d** **Difference in Means**	NHP B	11 WM sessions	Wilcoxon Signed Rank Test	Correct and incorrect	*Rank = 55* Correct mean = 288.26 **median** =277.23 Incorrect mean = 90.09 **median** = 71.43	0.002
**S4c** **Correlation analysis using task boundaries**	NHP B, NHP T	17 WM sessions	Paired T-Test	Correct and incorrect trials	*t* = *2.28* Correct **mean** = 0.56 median = 0.46 Incorrect **mean** = 0.39 median = 0.38	0.04

SUPPLEMENTARY FIGURE STATISTICS						
**Fig.**	**Subj.**	**Data**	**Stat Test**	**Comparison**	**Stat-Value**	**P-Value**
**S4d**<br>**Correlation analysis using task boundaries**	NHP B, NHP T	12 WM sessions, 12 perception sessions	Paired T-Test	WM task versus perception-guided navigation task	*t* = *5.10*<br><br>WM<br>**mean** = 0.65<br>median = 0.66<br>Perception<br>**mean** = 0.38<br>median = 0.36	3.4180E-04

SUPPLEMENTARY FIGURE STATISTICS						
**Fig.**	**Subj.**	**Data**	**Stat Test**	**Comparison**	**Stat-Value**	**P-Value**
**S4e**<br><br>**Ablation test - correct trials**	NHP B, NHP T	17 WM Sessions	1-way ANOVA + Tukey-Kramer post hoc<br><br>10-20<br>-30<br>-40<br>-50<br>-60<br>-70<br>-80<br>-90<br><br>20-30<br>-40<br>-50<br>-60<br>-70<br>-80<br>-90<br><br>30-40<br>-50<br>-60<br>-70<br>-80<br>-90<br><br>40-50<br>-60<br>-70<br>-80<br>-90<br><br>50-60<br>-70<br>-80<br>-90<br><br>60-70<br>-80<br>-90<br><br>70-80<br>-90<br><br>80-90	Correlation analysis after randomly removing a percentage of cells. (Mean across 10 iterations for each session is plotted.)	F(8,144) = 5.48<br>10<br>**mean** = 0.60<br>median = 0.62<br>20<br>**mean** = 0.58<br>median = 0.62<br>30<br>**mean** = 0.57<br>median = 0.59<br>40<br>**mean** = 0.56<br>median = 0.58<br>50<br>**mean** = 0.53<br>median = 0.55<br>60<br>**mean** = 0.50<br>median = 0.50<br>70<br>**mean** = 0.43<br>median = 0.44<br>80<br>**mean** = 0.39<br>median = 0.36<br>90<br>**mean** = 0.33<br>median = 0.32	4.8797e-06<br><br>1.0000<br>0.9999<br>0.9993<br>0.9590<br>0.7677<br>0.0757<br>**0.0069**<br>**0.0001**<br><br>1.0000<br>1.0000<br>0.9971<br>0.9400<br>0.2026<br>**0.0273**<br>**0.0006**<br><br>1.0000<br>0.9989<br>0.9632<br>0.2517<br>**0.0377**<br>**0.0009**<br><br>0.9998<br>0.9848<br>0.3389<br>0.0600<br>**0.0017**<br><br>0.9999<br>0.7032<br>0.2303<br>**0.0135**<br><br>0.9339<br>0.5244<br>**0.0616**<br><br>0.9983<br>0.7229<br><br>0.9857

SUPPLEMENTARY FIGURE STATISTICS						
**Fig.**	**Subj.**	**Data**	**Stat Test**	**Compariso n**	**Stat-Value**	**P-Value**
**S4f**<br><br>**Correlation analysis**	NHP B, NHP T	17 WM sessions	1-way ANOVA<br>Tukey-Kramer post hoc	"Target" location vs "optimal" trajectories vs movement trajectories in VR "world" coordinates vs visuospatial "screen" trajectories.<br><br>Target-optimal<br>Target-world<br>Target-Screen<br>Optimal-World<br>Optimal-Screen<br>World-Screen	*F(3,64)= 4.29*<br><br>Targets<br>**mean** = 0.46<br>median =<br>Optimal<br>**mean** = 0.46<br>median = 0.36<br>World<br>**mean** = 0.46<br>median =<br>Screen<br>**mean** = 0.61<br>median = 0.36	0.008<br><br>1<br>1<br>0.02<br>0.99<br>0.02<br>0.03
**S7b**<br><br>**Epoch Decoding**	NHP B, NHP T	17 WM sessions	Paired T-Test	Compare to chance (33%)	t = 12.4181<br>Combined Accuracy<br>mean=79%<br>median=88%<br><br>NHP T<br>**mean**=60%<br>median=60%<br><br>NHP B<br>**mean**=90%<br>median=90%	1.2507E-09

SUPPLEMENTARY FIGURE STATISTICS

Fig.	Subj.	Data	Stat Test	Comparison	Stat-Value	P-Value
**S7c**<br>**Decoding using task boundaries**	NHP B, NHP T	17 WM sessions	T-Test	Row decoding vs chance (33%)<br><br>Column decoding vs chance (33%)<br><br>Target decoding vs chance (11%)	t = 8.9743<br>Row **mean**= 48% median= 47%<br><br>t = 7.7598<br>Column **mean**= 66% median= 74%<br><br>t = 8.1505<br>Target **mean**= 31% median= 30%	1.2109E-07<br><br>8.2176e-07<br><br>4.3509e-07
**S8e**<br>**ODR**<br>**Difference in means**	NHP B, NHP T	17 WM sessions<br><br>12 ODR Sessions	Kruskal Wallis<br><br>Tukey Kramer Post-Hoc	VR and ODR<br><br>VR-ODR1<br>VR-ODR2<br>ODR1-ODR2	*H(2,34)* = 27.2<br><br>VR mean=253.98 **median**=270.9<br>ODR1 mean=91.53 **median**=93.2<br>ODR2 mean=34.85 **median**=31.6	1.2E-06<br><br>0.01<br>1.02E-06<br>0.29

SUPPLEMENTARY FIGURE STATISTICS						
**Fig.**	**Subj.**	**Data**	**Stat Test**	**Comparison**	**Stat-Value**	**P-Value**
**S9a** **Percent correct**	NHP T	8 Ketamine Sessions	2-way ANOVA	Injection period drug (ketamine, saline)	Drug: $F(1,30)=2.8$	0.107
					Injection Period: $F(2,30)=2.9$	0.066
					Interaction: $F(2,30)=2.2$	0.135
			Tukey Kramer Post Hoc	Ket pre-injection, Sal pre-injection		0.894
				Ket pre-injection, Ket early post-injection		0.037
				Ket pre-injection, Sal early post-injection		1
				Ket pre-injection, Ket late post-injection		0.961
				Ket pre-injection, Sal late post-injection		0.864
				Sal pre-injection, Ket early post-injection		0.027
				Sal pre-injection, Sal early post-injection		0.972
				Sal pre-injection, Ket late post-injection		0.581

SUPPLEMENTARY FIGURE STATISTICS						
**Fig.**	**Subj.**	**Data**	**Stat Test**	**Comparison**	**Stat-Value**	**P-Value**
				Sal pre-injection, Sal late post-injection	**<u>Ketamine</u>**<br><u>Pre</u><br>**mean**=54, median=60<br><u>Early-Post</u><br>**mean**=30, median=24<br><u>Late-Post</u><br>**mean**=47, median=56<br>**<u>Saline</u>**<br><u>Pre</u><br>**mean**=65, median=70<br><u>Early-Post</u><br>**mean**=55, median=56<br><u>Late-Post</u><br>**mean**=42, median=57	0.487
				Ket early post-injection, Sal early post-injection		0.198
				Ket early post-injection, Ket late post-injection		0.205
				Ket early post-injection, Sal late post-injection		0.873
				Sal early post-injection, Ket late post-injection		0.980
				Sal early post-injection, Sal late post-injection		0.904
				Ket late post-injection, Sal late post-injection		0.993

SUPPLEMENTARY FIGURE STATISTICS						
**Fig.**	**Subj.**	**Data**	**Stat Test**	**Comparison**	**Stat-Value**	**P-Value**
**S9b** **Percent Correct**	NHP B	9 Ketamine sessions	2-way ANOVA	Injection period drug (ketamine, saline)	Drug: *F*(1,33)=12.9	0.001
					Injection Period: *F*(2,33)=6.7	0.0034
					Interaction: *F*(2,33)=9.5	0.0005
			Tukey Kramer Post-Hoc	Ket pre-injection, Sal pre-injection		1
				Ket pre-injection, Ket early post-injection		0
				Ket pre-injection, Sal early post-injection		0.99
				Ket pre-injection, Ket late post-injection		0.76
				Ket pre-injection, Sal late post-injection		0.988
				Sal pre-injection, Ket early post-injection		0.0001
				Sal pre-injection, Sal early post-injection		1
				Sal pre-injection,Ket late post-injection		0.827

SUPPLEMENTARY FIGURE STATISTICS						
**Fig.**	**Subj.**	**Data**	**Stat Test**	**Comparison**	**Stat-Value**	**P-Value**
				Sal pre-injection, Sal late post-injection	**Ketamine**<br>Pre<br>**mean**=89, median=90<br>Early-Post<br>**mean**=39, median=43<br>Late-Post<br>**mean**=80, median=83<br>**Saline**<br>Pre<br>**mean**=91, median=90<br>Early-Post<br>**mean**=93, median=92<br>Late-Post<br>**mean**=84, median=84	0.98
				Ket early post-injection, Sal early post-injection		0
				Ket early post-injection, Ket late post-injection		0.0001
				Ket early post-injection, Sal late post-injection		0.0006
				Sal early post-injection, Ket late post-injection		0.74
				Sal early post-injection, Sal late post-injection		0.96
				Ket late post-injection, Sal late post-injection		0.1

SUPPLEMENTARY FIGURE STATISTICS						
**Fig.**	**Subj.**	**Data**	**Stat Test**	**Comparison**	**Stat-Value**	**P-Value**
**S9c** **Difference in Means**	NHP T	7 Ketamine Sessions	Kruskal Wallis	Injection period	*H(2,17) = 3.46* Pre mean=137.7, **median**=94.9 Early-Post mean=9.6, **median**=0.59 Late-Post mean=34.98, **median**=70.09	0.1775
**S9d** **Difference in Means**	NHP B	9 Ketamine Sessions	Kruskal Wallis Tukey Kramer Post-Hoc	Injection Period Pre, early-post Pre, late-post Early-post, late-post	*H(2,24)* = 7.24 Pre mean=180.47, **median**=187.5 Early-Post mean=68.8, **median**=44.63 Late-Post mean=104.88, **median**=100.8	0.03 0.02 0.24 0.53

SUPPLEMENTARY FIGURE STATISTICS						
**Fig.**	**Subj.**	**Data**	**Stat Test**	**Compariso n**	**Stat-Value**	**P-Value**
**S9e** **Correlation analysis** **Neural Boundaries**	NHP T	7 Ketamine Sessions	1-way ANOVA	Injection Period	F*(2,24) = 0.82* Pre **mean**= 0.32 median= 0.30 Early-Post **mean**= 0.28 median= 0.25 Late-Post **mean**= 0.36 median= 0.33	0.45
**S9f** **Correlation analysis** **Neural Boundaries**	NHP B	9 Ketamine Sessions	1-way ANOVA	Injection Period	F*(2,24) = 7.52* Pre **mean**= 0.62 median= 0.63 Early-Post **mean**= 0.32 median= 0.27 Late-Post **mean**= 0.47 median= 0.38	0.0029
			Tukey Kramer Post-Hoc	Pre, early-post		0.0020
				Pre, late-post		0.1345
				Early-post, late-post		0.1665

SUPPLEMENTARY FIGURE STATISTICS						
**Fig.**	**Subj.**	**Data**	**Stat Test**	**Compariso n**	**Stat-Value**	**P-Value**
**S9g**<br>**Correlation analysis**<br>**Task Boundaries**	NHP B, NHP T	16 Ketamine Sessions	1-way ANOVA<br><br>Tukey Kramer Post-Hoc	Injection Period<br><br>Pre, early-post<br>Pre, late-post<br>Early-post, late-post	F*(2,24) = 3.32*<br>Pre<br>**mean**= 0.51 median= 0.51<br>Early-Post **mean**= 0.35 median= 0.36<br>Late-Post **mean**= 0.44 median= 0.41	0.0451<br><br>0.0360<br>0.5484<br>0.2950
**S9h**<br>**Difference in Means (Saline)**	NHP B, NHP T	7 Saline Sessions	Kruskal Wallis	Injection Period	*H(2,18) = 2.07*<br><br>Pre mean=165.4 3, **median**=1 64.1<br>Early-Post mean=136.2 7, **median**=1 44.5<br>Late-Post mean=96.76, **median**=106 .6	0.36

SUPPLEMENTARY FIGURE STATISTICS						
**Fig.**	**Subj.**	**Data**	**Stat Test**	**Comparison**	**Stat-Value**	**P-Value**
**S9i**<br><br>**Correlation Analysis (Saline)**<br><br>**Task Boundaries**	NHP B, NHP T	7 Saline Sessions	1-way ANOVA<br><br>Tukey Kramer Post-Hoc	Injection Period<br><br>Pre, early-post<br><br>Pre, late-post<br><br>Early-post, late-post	F*(2,18) = 0.34*<br><br>Pre<br><br>**mean**= 0.46 median= 0.36<br><br>Early-Post **mean**= 0.44 median= 0.40<br><br>Late-Post **mean**= 0.38 median= 0.39	0.7181

ADDITIONAL STATISTICS						
**Fig.**	**Subj.**	**Data**	**Stat Test**	**Comparison**	**Stat-Value**	**P-Value**
**Observed correlations compared to chance**	NHP B, NHP T	17 WM sessions	Paired T-Test	Neural boundary delay sequence-trajectory correlation compared to mean of shuffled null obtained by shuffling target labels 100 times.	t = -4.23<br><br>Observed:<br>**mean=** 0.5440<br>median= 0.4965<br><br>Shuffled:<br>**mean=** 0.3227<br>median= 0.3190	6.31E-04
**Single vs multiple contributions**	NHP B, NHP T	17 WM sessions	Paired T-Test	Correlation analysis in which each cell can contribute to the sequence for each epoch, to test the assumption that each cell contributes only once to a single trial sequence.Here, the correlation of neural boundary delay sequences are considered.	*t =0.2473*<br><br>Single<br>**mean** = 0.54<br>median =0.50<br><br>Multiple<br>**mean** = 0.55<br>median =0.62	0.08
**Percent of cells that are "time selective"**	NHP B, NHP T	17 WM sessions	WM:<br>mean=4.8%, median=4.7%<br>Perception:<br>mean=6.2%, median=3.4%<br>PreKet:<br>mean=5.0%, median=3.6%<br>PostKet:<br>mean=2.8%, median=2.3%<br>LatePost Ket:<br>mean=3.5%, median=2.6%			

ADDITIONAL STATISTICS						
**Fig.**	**Subj.**	**Data**	**Stat Test**	**Comparison**	**Stat-Value**	**P-Value**
**Delay-Navigation sequence correlation**	NHP B, NHP T	17 WM sessions	Test whether observed absolute correlation value of delay sequence to same-trial navigation sequence is larger than the 99th percentile of correlation values between the delay sequence and 25 randomly drawn different trial navigation sequences.	Sequences from same trial vs from different trials. Absolute value correlation from delay sequences to 1) same-trial nav sequences, vs 2) mean across different-trial nav sequences.		Delay sequences are less correlated to same-trial navigation sequences than different-trial navigation sequences on 96.31% of trials.
