# Supplementary material for: Neuronal activation sequences in lateral prefrontal cortex encode visuospatial working memory during virtual navigation
Source: Nat Commun. 2024 May 25;15:4471. doi: 10.1038/s41467-024-48664-9 (PMC11127969; doi:10.1038/s41467-024-48664-9)
Supplement: Supplementary file 3 — Description of Additional Supplementary Files [file 41467_2024_48664_MOESM3_ESM.pdf]

Description of Additional Supplementary Files

Supplementary Movie 1: An example trial of the WM task can be found here: https://www.youtube.com/watch?v=nZDYJw2aFLQ Note that the video is played back at an increased speed. During the trial, a cue location (red vertical bar) is presented for 1 second, then disappears for a 2 second memory delay, after which the subject responds by navigating to the remembered location using a joystick.
