# Supplementary material for: Neuronal activation sequences in lateral prefrontal cortex encode visuospatial working memory during virtual navigation
Source: Nat Commun. 2024 May 25;15:4471. doi: 10.1038/s41467-024-48664-9 (PMC11127969; doi:10.1038/s41467-024-48664-9)
Supplement: Supplementary file 5 — Reporting Summary [file 41467_2024_48664_MOESM5_ESM.pdf]

Corresponding author(s): Lyle Muller and Julio Martinez-Trujillo

Last updated by author(s): Feb 8 2024

Reporting Summary

Nature Portfolio wishes to improve the reproducibility of the work that we publish. This form provides structure for consistency and transparency in reporting. For further information on Nature Portfolio policies, see our Editorial Policies and the Editorial Policy Checklist.

Statistics

For all statistical analyses, confirm that the following items are present in the figure legend, table legend, main text, or Methods section.

n/a	Confirmed	
☐	☒	The exact sample size (*n*) for each experimental group/condition, given as a discrete number and unit of measurement
☒	☐	A statement on whether measurements were taken from distinct samples or whether the same sample was measured repeatedly
☐	☒	The statistical test(s) used AND whether they are one- or two-sided<br>*Only common tests should be described solely by name; describe more complex techniques in the Methods section.*
☐	☒	A description of all covariates tested
☐	☒	A description of any assumptions or corrections, such as tests of normality and adjustment for multiple comparisons
☐	☒	A full description of the statistical parameters including central tendency (e.g. means) or other basic estimates (e.g. regression coefficient) AND variation (e.g. standard deviation) or associated estimates of uncertainty (e.g. confidence intervals)
☐	☒	For null hypothesis testing, the test statistic (e.g. *F*, *t*, *r*) with confidence intervals, effect sizes, degrees of freedom and *P* value noted<br>*Give P values as exact values whenever suitable.*
☒	☐	For Bayesian analysis, information on the choice of priors and Markov chain Monte Carlo settings
☒	☐	For hierarchical and complex designs, identification of the appropriate level for tests and full reporting of outcomes
☐	☒	Estimates of effect sizes (e.g. Cohen's *d*, Pearson's *r*), indicating how they were calculated

Our web collection on statistics for biologists contains articles on many of the points above.

Software and code

Policy information about availability of computer code

Data collection	The virtual task environment was developed using Unreal Engine 3 development kit, utilizing Kismet sequencing and UnrealScript (UDK, May 2012 release; Epic Games). Details about this platform and the recording setup can be found in Doucet et al., Journal of Neuroscience Methods 266, 2016. Electrophysiology data were collected using a Cerebus Neuronal Signal Processor (Blackrock Microsystems) via a Cereport adapter. The extracted spikes were semi-automatically sorted using Plexon Offline Sorter (Plexon Inc.) and then manually supervised.
Data analysis	Custom MATLAB code was used for data analysis in this study (R2021a).

For manuscripts utilizing custom algorithms or software that are central to the research but not yet described in published literature, software must be made available to editors and reviewers. We strongly encourage code deposition in a community repository (e.g. GitHub). See the Nature Portfolio guidelines for submitting code & software for further information.

Data

Policy information about availability of data

All manuscripts must include a data availability statement. This statement should provide the following information, where applicable:

- Accession codes, unique identifiers, or web links for publicly available datasets
- A description of any restrictions on data availability
- For clinical datasets or third party data, please ensure that the statement adheres to our policy

The processed data that support these findings will be made available at this study's GitHub repository:
http://github.com/mullerlab/buschEAsequences
The raw data that support the findings of this study are available from the corresponding author upon reasonable request.

Research involving human participants, their data, or biological material

Policy information about studies with human participants or human data. See also policy information about sex, gender (identity/presentation), and sexual orientation and race, ethnicity and racism.

Reporting on sex and gender	N/A
Reporting on race, ethnicity, or other socially relevant groupings	N/A
Population characteristics	N/A
Recruitment	N/A
Ethics oversight	N/A

Note that full information on the approval of the study protocol must also be provided in the manuscript.

Field-specific reporting

Please select the one below that is the best fit for your research. If you are not sure, read the appropriate sections before making your selection.

☒ Life sciences ☐ Behavioural & social sciences ☐ Ecological, evolutionary & environmental sciences

For a reference copy of the document with all sections, see nature.com/documents/nr-reporting-summary-flat.pdf

Life sciences study design

All studies must disclose on these points even when the disclosure is negative.

Sample size	Two male macaque monkeys were used for this study. No sample size calculation was performed. The number used conforms to standards in the field for non-human primate electrophysiology experiments. No effects of sex have been reported on the variables of interest.
Data exclusions	All experimental sessions were included in this study. Individual neurons were required to exhibit firing rates above 0.5 Hz.
Replication	All of the reported findings in one monkey were replicated using similar methodology in the second monkey.
Randomization	Randomization is not relevant for this study as there are no experimental groups.
Blinding	Blinding was not relevant to this study as no experimental group was defined.

Reporting for specific materials, systems and methods

We require information from authors about some types of materials, experimental systems and methods used in many studies. Here, indicate whether each material, system or method listed is relevant to your study. If you are not sure if a list item applies to your research, read the appropriate section before selecting a response.

Materials & experimental systems

n/a	Involved in the study	
☒	☐	Antibodies
☒	☐	Eukaryotic cell lines
☒	☐	Palaeontology and archaeology
☐	☒	Animals and other organisms
☒	☐	Clinical data
☒	☐	Dual use research of concern
☒	☐	Plants

Methods

n/a	Involved in the study	
☒	☐	ChIP-seq
☒	☐	Flow cytometry
☒	☐	MRI-based neuroimaging

Animals and other research organisms

Policy information about studies involving animals; ARRIVE guidelines recommended for reporting animal research, and Sex and Gender in Research

Laboratory animals	Two adult male rhesus macaques (Macaca mulatta) were used in this study (10 and 9 years of age).
Wild animals	The study did not involve wild animals.
Reporting on sex	The experiments in this study involved a total of two subjects, following our obligation to reduce the number of animals used. The sex of each animal has been recorded to facilitate future meta-analyses across studies, in order to understand potential sex-based differences in working memory behavior.
Field-collected samples	The study did not involve samples collected from the field.
Ethics oversight	Animal care and handling (i.e., basic care, animal training, surgical procedures, and experimental injections) were pre-approved by the University of Western Ontario Animal Care Committee. This approval ensures that federal (Canadian Council on Animal Care), provincial (Ontario Animals in Research Act), regulatory bodies (e.g., CIHR/NSERC), and other national standards (CALAM) for the ethical use of animals are followed. The oculomotor delayed response task experiment complied with Canadian policies and regulations and was preapproved by the McGill University Animal Care Committee (Leavitt, 2017b, Leavitt, 2018).

Note that full information on the approval of the study protocol must also be provided in the manuscript.

Plants

Seed stocks	N/A
Novel plant genotypes	N/A
Authentication	N/A
